# Center-of-Gravity-Aware Graph Convolution for Unsafe Behavior Recognition of Construction Workers

**DOI:** 10.3390/s25175493

**Published:** 2025-09-04

**Authors:** Peijian Jin, Shihao Guo, Chaoqun Li

**Affiliations:** School of Emergency Science and Engineering, Jilin Jianzhu University, 5088 Xincheng Avenue, Nanguan District, Changchun 130119, China

**Keywords:** human action recognition, construction safety, spatio-temporal graph convolutional network

## Abstract

Falls from height are a critical safety concern in the construction industry, underscoring the need for effective identification of high-risk worker behaviors near hazardous edges for proactive accident prevention. This study aimed to address this challenge by developing an improved action recognition model. We propose a novel dynamic spatio-temporal graph convolutional network (CoG-STGCN) that incorporates a center of gravity (CoG)-aware mechanism. The method computes global and local CoG using anthropometric priors and extracts four key dynamic CoG features, which a Multi-Layer Perceptron (MLP) then uses to generate modulation weights that dynamically adjust the skeleton graph’s adjacency matrix, enhancing sensitivity to stability changes. On a self-constructed dataset of eight typical edge-related hazardous behaviors, CoG-STGCN achieved a Top-1 accuracy of 95.83% (baseline ST-GCN: 93.75%) and an average accuracy of 94.17% in fivefold cross-validation (baseline ST-GCN: 92.91%), with significant improvements in recognizing actions involving rapid CoG shifts. The CoG-STGCN provides a more effective and physically informed approach for intelligent unsafe behavior recognition and early warning in built environments.

## 1. Introduction

The construction industry suffers from a high incidence of accidents, with falls from height being particularly frequent and lethal [1]. Beyond inadequate safety facilities, a primary cause is the unsafe behavior of workers. Current reliance on manual safety inspections is insufficient, hindered by limited coverage, poor real-time performance, and subjectivity. Therefore, developing technologies for real-time identification and intervention of such hazardous behaviors is crucial for improving construction safety.

Early technological solutions relied on traditional methods. For instance, Lim et al. [2] used BLE and RFID sensors to monitor worker location and movement, while Zhu et al. [3] employed visual detection with HOG features for tracking. While effective for specific tasks, these early approaches often lacked real-time processing capabilities and suffered from poor generalization. Recent advances in deep learning offer new solutions through video-based human action recognition (HAR). HAR primarily utilizes two data modalities: RGB images and skeleton data. Although information-rich, RGB-based methods lack robustness in complex environments like construction sites and are computationally intensive, hindering real-time performance [4]. In contrast, skeleton-based recognition focuses on human motion, making it resilient to background and lighting variations. This modality effectively captures temporal dynamics and naturally represents the human body as a topological graph, with joints as nodes and bones as edges. The pioneering ST-GCN model by Yan et al. [5] successfully applied Graph Convolutional Networks (GCNs) to this domain, inspiring much subsequent research and demonstrating strong performance in HAR tasks, including applications in building safety [6,7,8].

However, existing GCN-based methods have key limitations when applied to fall risk detection. Falls are intrinsically linked to a loss of balance and shifts in the body’s center of gravity (CoG). Most models rely on predefined skeletal connections or learn feature-level correlations, failing to explicitly model the dynamics of the CoG. This oversight hinders their ability to detect subtle motion patterns indicative of instability. To address this limitation, we propose the center-of-gravity-aware spatio-temporal graph convolutional network (CoG-STGCN). Its core innovation is a dynamic adjacency matrix that adjusts joint connection strengths based on real-time estimates of global and local CoG positions and velocities. This allows CoG-STGCN to more accurately capture motion features related to equilibrium changes, thus improving the identification of unsafe behaviors that may lead to falls.

The main contributions of this paper include the following:

1. We propose a novel center-of-gravity-aware spatio-temporal graph convolutional network (CoG-STGCN). The model addresses the shortcomings of existing methods by explicitly incorporating CoG dynamics into the graph construction process.
2. We conduct a systematic study to identify unsafe behaviors that lead to fall accidents in high-risk areas of construction sites (e.g., floor edges, openings).
3. We identify, define, and categorize a set of high-risk unsafe behaviors based on their impact on human balance. This provides a specific basis for risk assessment in such scenarios.

## 2. Related Works

### 2.1. Human Pose Estimation Model

Human pose estimation (HPE) methodologies are predominantly categorized into two main paradigms: bottom-up and top-down. The bottom-up approach begins by detecting all human keypoints across the entire image. Subsequently, techniques like Part Affinity Fields (PAFs) are employed to associate these keypoints, grouping them to form complete individual skeletons. Representative models adopting this strategy include OpenPose [9], HigherHRNet [10], and PersonLab [11]. While this approach offers advantages in speed for crowded, multi-person scenes, its accuracy and performance can be suboptimal in scenarios with fewer individuals compared to top-down methods.

In contrast, the top-down strategy first detects persons in the image using an object detector (e.g., Faster R-CNN [12], YOLO [13], SSD [14]) to generate bounding boxes for each individual. A single-person pose estimator is then applied within each bounding box to predict the keypoints. Notable algorithms in this category include AlphaPose [15], HRNet [16], and Mask R-CNN [17]. This two-stage process generally yields high accuracy, but it suffers from slower inference speeds in multi-person settings, and its performance is inherently dependent on the initial human detection stage.

To overcome the trade-offs between speed and accuracy inherent in these two paradigms, researchers have sought to develop more streamlined, end-to-end solutions. Inspired by the success of single-stage object detectors like the YOLO (You Only Look Once) family, models such as YOLO-Pose [18] have emerged. The core concept is to unify human detection and keypoint prediction into a single, deep neural network. This network performs a single forward pass to directly output bounding boxes and their corresponding pose information for all detected persons. This end-to-end architecture eliminates the bottlenecks of multi-stage pipelines. Specifically, YOLOv8-Pose has demonstrated performance that matches or exceeds previous methods in both accuracy and speed, particularly in multi-person scenarios. Its simplicity and efficiency make it a state-of-the-art front-end solution for applications requiring real-time, accurate skeleton data extraction, such as the behavioral analysis on construction sites targeted in our research.

### 2.2. Skeleton-Based Human Action Recognition

Early research in skeleton-based action recognition heavily relied on hand-crafted features. These features, such as the relative positions, angles, and velocities of joints, were manually designed and fed into traditional machine learning classifiers. However, these methods required significant domain expertise, exhibited limited generalization capabilities, and struggled to capture the complex spatio-temporal dynamics embedded in human actions.

With the proliferation of deep learning, research shifted towards methods that could automatically learn features from raw skeleton data. Initial attempts utilized Recurrent Neural Networks (RNNs) and variants like LSTM and GRU to model the temporal dynamics of action sequences [19]. While effective at capturing temporal dependencies, RNNs are deficient in modeling the inherent spatial graph structure of the human skeleton. Another approach involved transforming skeleton data into pseudo-images, allowing for feature extraction with Convolutional Neural Networks (CNNs) [20]. Although CNNs are powerful for image processing, forcing skeleton data—which have a natural graph structure—into a grid-like format can disrupt or discard the intrinsic physical and kinematic relationships between joints, leading to information loss.

To more effectively process the non-Euclidean structure of skeleton data, Graph Convolutional Networks (GCNs) were introduced and have since become the dominant paradigm. The seminal ST-GCN model innovatively represents a skeleton sequence as a spatio-temporal graph, where nodes correspond to joints and edges represent both spatial connections within a frame and temporal connections across frames. By alternately applying spatial and temporal convolutions, the model learns both spatial configuration and temporal dynamics simultaneously.

The success of ST-GCN has spurred extensive follow-up research aimed at enhancing its performance, with a significant focus on optimizing the graph structure. The original ST-GCN uses a fixed, predefined adjacency matrix, which limits its ability to capture dynamic, action-specific inter-joint relationships. To address this, subsequent works have proposed data-driven adaptive graph structures. For example, 2s-AGCN [21] learns an adaptive adjacency matrix to complement the physical skeleton graph, enabling the model to discover latent joint correlations. MS-G3D [22] introduced a multi-scale graph convolution module to capture dependencies at different ranges, while CTR-GCN [23] proposed a channel-wise topology modeling module for finer-grained interactions.

Despite achieving excellent performance, these advanced adaptive graph methods often overlook fundamental physical principles governing human motion. Specifically, they lack an explicit mechanism to account for the body’s center of gravity, a critical factor for maintaining balance and stability. This omission is a notable limitation, especially for recognizing high-risk behaviors like falls. Therefore, this paper aims to improve upon the ST-GCN framework by introducing a center-of-gravity-aware mechanism, designed to more effectively identify potential fall-risk behaviors.

## 3. Methods

### 3.1. Overall Framework

**Figure 1:**
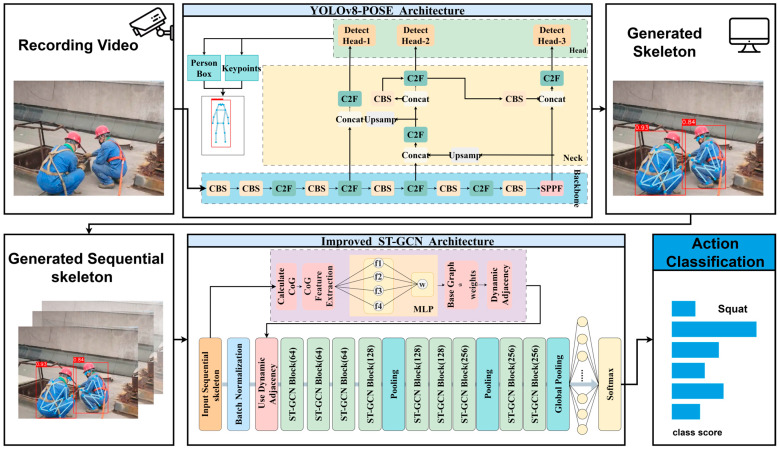
Flowchart of the system for detecting dangerous behavior in edge areas. The asterisk (*) denotes the element-wise multiplication.

The comprehensive architecture of our proposed system, designed to process video input through to final behavior classification, is illustrated in Figure 1. The workflow begins with raw video clips from on-site surveillance cameras, which are fed into the YOLOv8-Pose front-end module. This module efficiently generates a precise 2D human skeleton sequence for each frame. To capture the temporal dynamics of actions, the sequential skeleton data are then passed to our improved CoG-STGCN model. Herein lies our core innovation: the model first calculates the center of gravity (CoG) from the input skeletons and extracts CoG-related features. These features are processed by a Multi-Layer Perceptron (MLP) to generate a CoG-aware weight matrix, which is then combined with the base graph structure to create a dynamic adjacency matrix. This dynamic graph is subsequently used within a series of ST-GCN blocks to learn spatio-temporal features that are highly sensitive to body stability. The framework concludes with a Softmax classifier that outputs the final action classification.

### 3.2. YOLOv8-Pose

**Figure 2:**
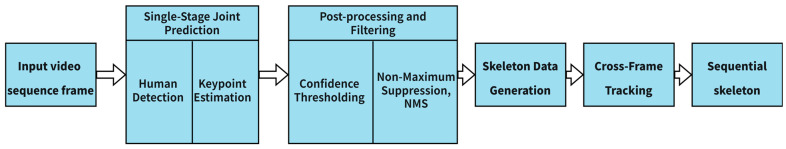
The overall pipeline for YOLOv8-Pose.

In order to efficiently and accurately extract the key points of the human skeleton from the input video stream, this paper chooses to use a single-stage pose estimation algorithm, YOLOv8-Pose, whose processing flow of the image is shown in Figure 2.

**Figure 3:**
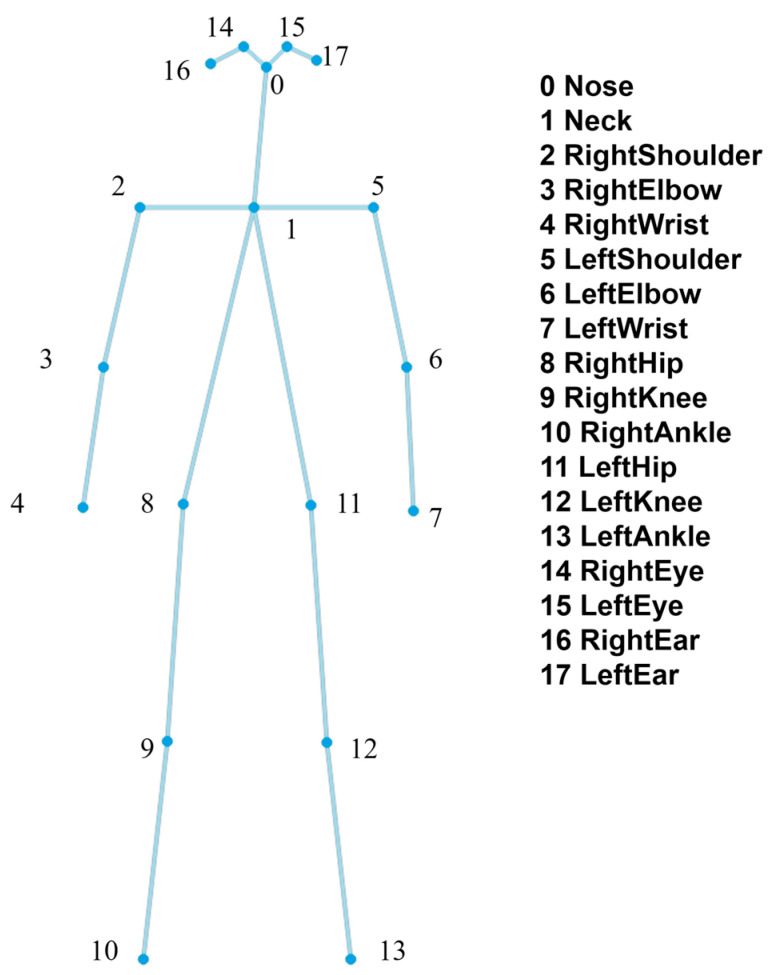
The definition of the 18-keypoint human skeleton.

Ultralytics provides pre-training models with different computational complexities and prediction accuracies for YOLOv8-Pose, and we choose a pre-training model for YOLOv8-Pose, taking into account the possibility of future deployment on resource-constrained edge devices and the priority of ensuring real-time processing. Considering the potential for future deployment on resource-constrained edge devices and prioritizing real-time processing, we chose the YOLOv8s-pose.pt pre-training model. The model is able to directly output the bounding box of each detected human instance as well as the 2D coordinates and confidence level of the standard 17 keypoints of COCO. In order to adapt to the input of the ST-GCN model, which usually requires 18 keypoints of the human skeleton, we generate the 18th keypoint, i.e., the joints of the neck, based on the keypoints of the left and right shoulders in the COCO format, and the distribution of the joints of the skeleton in the model is shown in Figure 3. To process continuous video frames and generate individual motion sequences, we utilized the integrated tracking functionality provided by the Ultralytics YOLOv8 framework and chose ByteTrack as the underlying tracking algorithm. Table 1 compares the performance of some mainstream pose estimation models, and YOLOv8s-pose is a more lightweight model while maintaining a high AP@50 (85.8%). It is worth noting that the total system overhead of AlphaPose as a Top-Down method also needs to account for the additional human body detector, whereas YOLOv8s-pose is an all-in-one design.

### 3.3. The Proposed COG-STGCN Model

#### 3.3.1. ST-GCN Model

**Figure 4:**
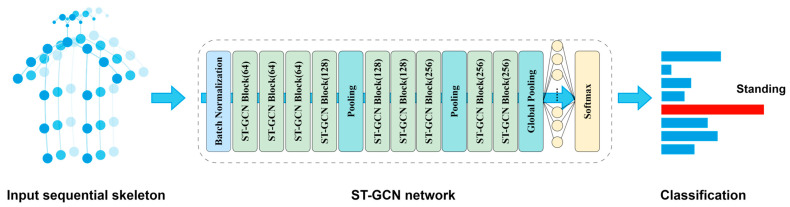
Workflow diagram of ST-GCN behavior recognition model.

In this study, we improve the ST-GCN model to enhance the recognition of dangerous behaviors in the adjacent area of a construction site. ST-GCN models the human skeleton sequence as a spatio-temporal graph G = (V, E), where node V represents the human body joints, and the edge E represents the skeletal connections and the interframe connections. ST-GCN learns spatial-temporal features by alternately applying spatial and temporal graph convolutions. The working principle and flow of the model are shown in Figure 4, which receives the time series data of the skeleton joint points and constructs them into a spatio-temporal graph, then learns the spatial structural features and temporal dynamic features of the actions alternately through multiple spatio-temporal graph convolution modules, and finally aggregates the information through global pooling and outputs the final action categories using classifiers.

The spatial graph convolution operation of this model can be formulated as

(1) $$f_{out}=\sum_{k}^{K_{v}}(\Lambda_{k}^{-\frac{1}{2}}A_{k}\Lambda_{k}^{-\frac{1}{2}}f_{in}{)W}_{k}⊙M_{k}$$

where $f_{in}$ and $f_{out}$ are the input and output features, respectively. $K_{v}$ is the kernel size in the spatial dimension, which is set to 3, $W_{k}$ is the learnable weight matrix, and $A_{k}$ is the predefined adjacency matrix representing the graph’s connectivity structure. $\Lambda_{k}^{ii}=\sum_{j}\left(A_{k}^{ij}\right)+\alpha$ is the corresponding degree matrix, where α is set to 0.001 to avoid empty rows in $A_{k}$ and $M_{k}$ is an attention map used to represent the importance of each vertex. As can be seen from Equation (1), the ST-GCN model utilizes a fixed, predefined adjacency matrix $A_{k}$. This matrix is constructed based on the physical connections of the human body and a spatial partitioning strategy, and it remains static for all input samples. This makes it difficult to adaptively adjust the graph structure and connection strengths according to the dynamic characteristics of the actions themselves. Consequently, a key limitation of ST-GCN in capturing specific complex actions lies in its use of a fixed, unchanging graph structure.

#### 3.3.2. Center-of-Gravity-Based Improvement to the ST-GCN Model

**Figure 5:**
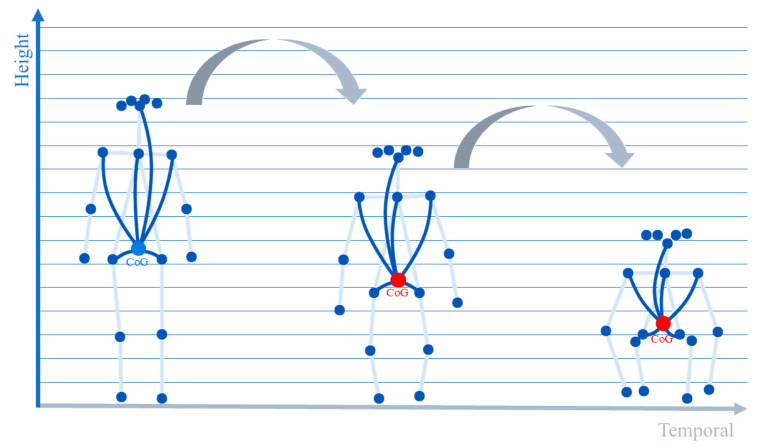
Schematic diagram of the change in center of gravity with the squatting movement.

In order to overcome the limitations of the ST-GCN model described in the previous section and enable it to capture real-time motion information more adequately, a dynamic adaptive graph structure updating rule needs to be found. In the field of biomechanics, the center of gravity of the human body is widely regarded as one of the core parameters for analyzing postural control and movement dynamics [24], which not only represents the centralized point of body mass distribution, but also the dynamic changes of its position, velocity, and acceleration are a high degree of generalization of the individual’s overall movement state and postural stability. For example, as shown in Figure 5, the center of gravity changes with the body posture, and the center of gravity of the character falls continuously in vertical height. The trajectory pattern of the CoG thus contains rich discriminative information. Therefore, we leverage the CoG, a physically meaningful metric, as the basis for dynamically adjusting the graph structure. Considering the close relationship between the position of the center of gravity and the position of each node of the human body, we adopt the a priori knowledge of anthropometry and weighted average of each part of the human body to introduce this information of CoG into the model. Specifically, we calculate the global center of gravity at each frame of the video sequence as follows:

(2) $$P_{COG}(t)=\sum_{v=1}^{V}w_{v}\cdot P_{v}(t)$$

where $P_{v_{\left(t\right)}}$ is the 2D coordinate of the *v*-th keypoint at frame *t*. $P_{COG_{\left(t\right)}}$ is the estimated CoG at frame *t*, calculated as a weighted sum of the keypoint coordinates. $w_{v}$ represents the mass weight assigned to the *v*-th keypoint. The weights were selected with reference to the percentage of mass of human segments determined in anthropometric and biomechanical studies (Table 2), and we approximated the mass share of segments such as head, torso, left/right arm, left/right leg, etc., by assigning them to the articulation points of the skeleton that best represent these segments, which is also reflected in Figure 5. These specific body mass distribution data references are derived from the segmental inertia parameters proposed by de Leva et al. [25] after adapting and summarizing classical anthropometric data, which are now widely used in the field of biomechanics.

In addition to calculating the global center of gravity, which represents the mass distribution of the whole body, in order to capture the motion state of each core region of the human body, we also designed a localized core center of gravity calculation method, and this localized center of gravity can help decouple the motion contribution of the torso and limbs and provide richer information. Considering the relatively even distribution of mass in the core region, its specific calculation formula is as follows:

(3) $$P_{Core}(t)=\frac{1}{\left|V_{Core}\right|}\sum_{v\in V_{Core}}P_{v_{Core}}(t)$$

where $P_{v_{Core\left(t\right)}}$ is the 2D coordinate of the *v*-th keypoint belonging to the core keypoint subset $V_{Core}$ at frame *t*, and $\left|V_{Core}\right|$ denotes the number of keypoints in the core region; for instance, the left arm region consists of three keypoints. $P_{Core_{\left(t\right)}}$ represents the 2D coordinate of the local CoG at frame *t*, which is obtained by calculating the arithmetic mean of all keypoints within the subset $V_{Core}$. The specific distribution of local centroids and the corresponding keypoint selections are detailed in Table 3.

With a formula that can approximately represent the position of the body’s center of gravity, in order to maximize the extraction of the information carried by the CoG, we performed feature construction based on the CoG, and obtained a total of four features: the distance of the node to the global center of gravity (f1), the rate of movement of the global center of gravity (f2), the distance of the node to the local center of gravity (f3), and the rate of movement of the local center of gravity (f4). Two distance features (f1, f3) are used to describe the spatial relationship between the node and the global and local regions, and two rate features (f2, f4) are used to describe the motion intensity of the body as a whole and the local region. In order to provide the subsequent dynamic graph modulation mechanism with more stable motion information over a certain time scale and to avoid the noise effect of transient frames, we adopt a feature extraction strategy similar to that of a time window. Specifically, we set a time window of length T frames, and we set T = 30 frames, and the center-of-gravity-related features described subsequently are all based on the aggregated features computed from the skeleton data within such a time window. The formulas for each feature are shown below:

(4) $$\mathrm{Dist}(P_{A},P_{B})=||P_{A}-P_{B}|{|}_{2}$$

(5) $$f_{1,v}=\frac{1}{T}\sum_{t=1}^{T}\mathrm{Dist}\left(P_{v}\left(t\right),P_{CoG}\left(t\right)\right)$$

Equation (4) defines a method for calculating the Euclidean distance between two points: $P_{A}$ and $P_{A}$. Equation (5) calculates the average Euclidean distance from the *v*-th joint point $P_{v}\left(t\right)$ to the global center of gravity $P_{CoG}\left(t\right)$ of the same frame within the time window of the current T-frame, where t represents a certain frame index within the time window.

(6) $$f_{3,v,l}=\frac{1}{T}\sum_{t=1}^{T}Dist\left(P_{v_{Core}}\left(t\right),P_{Core,l}\left(t\right)\right)$$

(7) $$F_{3,v}=(f_{3,v,1},f_{3,v,2},\ldots f_{3,v,l})\;,\;\;l=6$$

Equation (6) calculates the average Euclidean distance from the *v*-th joint point $P_{v_{Core}}\left(t\right)$ of each core region to the local center of gravity $P_{Core,l}\left(t\right)$ of that core region throughout the segment to capture the average relative spatial relationship between the parts within the core region of the body. $F_{3,v}$, shown in Equation (7), is a combination of the average distance feature $f_{3,v,l}$ from the joints of each core region within the window to its corresponding local center of gravity.

(8) $$f_{2}=\frac{1}{T}\sum_{t=1}^{T}\left||(P_{CoG}(t)-P_{CoG}(t-1)\right)|{|}_{2}$$

Equation (8) calculates the average rate of movement of the global center of gravity $P_{CoG}$, throughout the action segment, i.e., the magnitude of the global center of gravity displacements between each frame is summed and time averaged to measure the average degree of fastness of the overall body movement.

(9) $$f_{4,l}=\frac{1}{T}\sum_{t=1}^{T}||(P_{Core,l}(t-1)-P_{Core,l}(t)-(P_{CoG}(t-1)-P_{CoG}(t)))|{|}_{2}$$

(10) $$F_{4}=(f_{4,1},f_{4,2},\ldots f_{4,l})\;,\;\;l=6$$

Equation (9) calculates the magnitude of the average relative velocity of the local center of gravity $P_{Core,l}(t)$ of a particular core region moving with respect to the global center of gravity $P_{CoG}$ throughout the entire action segment to capture the average internal rate of motion of that core region with respect to the whole body overall motion. $F_{4}$, shown in Equation (10), consists of a combination of different core region local center of gravity relative rate features $f_{4}$.

Finally, for each joint, the feature vector of its input MLP is formed by stitching the four sets of previously computed features sequentially in feature dimensions. Thus, ${Input}_{v}$ is a composite feature vector of dimension 2 + 2L, which can be expressed by Equation (11):

(11) $${Input}_{v}=[f_{1,v},f_{2},F_{3,v}^{T},F_{4}^{T}{]}^{T}$$

This feature vector enables the MLP to perceive the overall body dynamics and spatial configuration of the input sample. Through backpropagation, the optimizer fine-tunes the network to understand the complex, non-linear relationships within the CoG features. The final output of the MLP is a tensor of shape (N, V, M), which generates a specific weight, w, for each node v. This weight signifies the degree to which the connections associated with that node should be preserved or weakened during the dynamic graph adjustment. This computation is performed independently for each person and sample, ensuring its applicability in multi-person scenarios.

**Figure 6:**
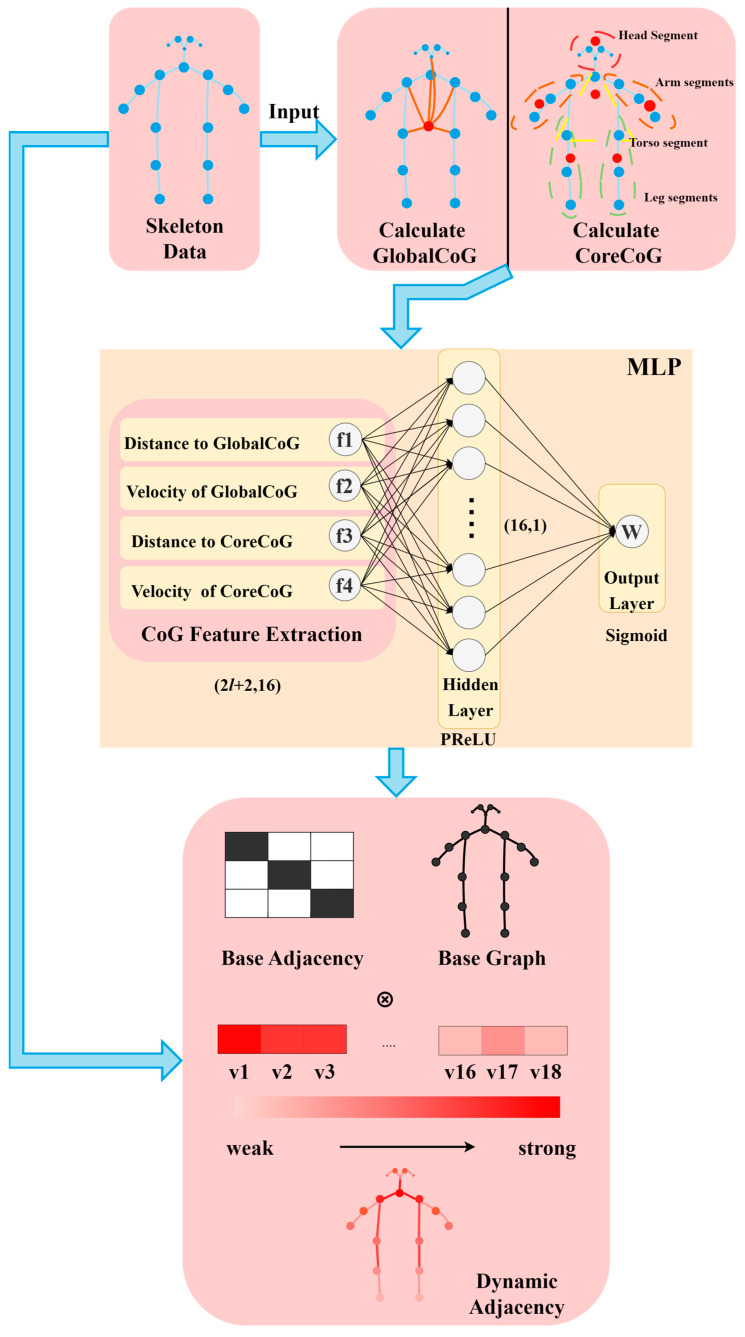
Flowchart of dynamic adjacency matrix generation based on center of gravity (CoG) feature extraction and MLP learning.

The process of generating this dynamic adjacency matrix is detailed in Figure 6. As illustrated in the figure, we define and compute two types of CoG from the input skeleton data: a global center of gravity (GlobalCoG), which represents the overall body balance, and a core center of gravity (CoreCoG), which focuses on the stability of the torso and trunk. Based on these two reference points, we extract four key features for each node: (f1) the distance to the GlobalCoG, (f2) the velocity of the GlobalCoG, (f3) the distance to the CoreCoG, and (f4) the velocity of the CoreCoG. These features form the input to the MLP, which then learns to generate the node-specific modulation weights, $W_{mlp}$.

The final dynamic adjacency matrix, $A_{cog,k}$, is then obtained by an element-wise product (Hadamard product) of the k-th subset of the base adjacency matrix, $A_{k}$, and the learned modulation weights:

(12) $$A_{cog,k}=A_{k}⊙W_{mlp}$$

This dynamically generated, sample-specific adjacency matrix is then used in the improved spatial graph convolution operation, which can be expressed as follows:

(13) $$f_{out}=\sum_{k}^{K_{v}}(\Lambda_{k}^{-\frac{1}{2}}A_{cog,k}\Lambda_{k}^{-\frac{1}{2}}f_{in}{)W}_{k}⊙M_{k}$$

It is worth noting that the overall architecture of CoG-STGCN follows the standard stacking pattern of multiple ST-GCN blocks. Our core improvement is the dynamic processing of the graph structure within each block. In scenarios where one or several keypoints required for CoG computation are occluded, the system degrades to using only the underlying fixed graph structure. This ensures a baseline level of performance and maintains the model’s robustness, preventing complete failure when faced with partial input data.

**Figure 7:**
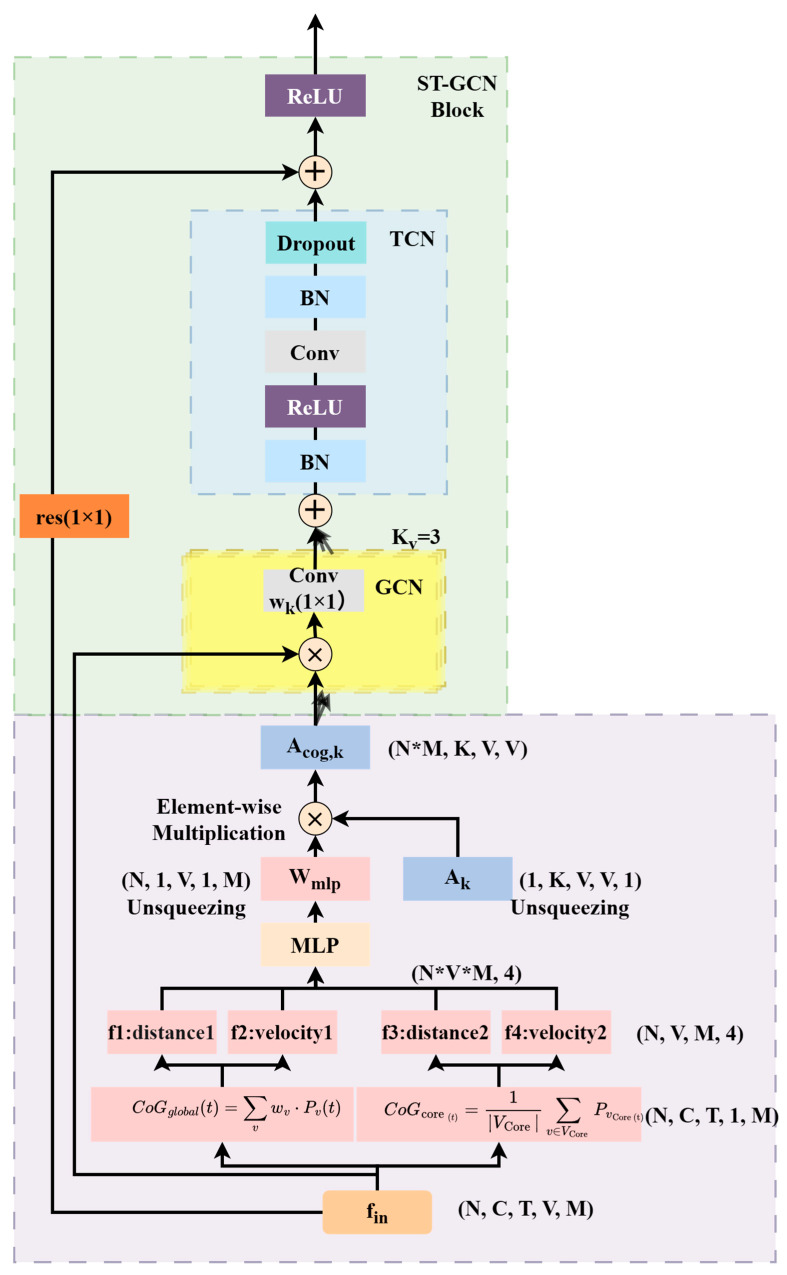
Architecture of the CoG-aware dynamic graph convolutional unit in CoG-STGCN.

The specific process is shown in Figure 7. The global center of gravity and each core center of gravity are computed from the input data, i.e., the 18-node skeleton sequence (N, C, T, V, M). Based on this center of gravity, we further extract four key CoG-related features), which together describe the overall stability and movement characteristics of the human body. The four features are fed into a multilayer perceptron (MLP) to learn the modulation weights W_mlp_ of the output nodes, which are expanded and shaped as (N, 1, V, 1, M) for tuning each node. The learned W_mlp_ is multiplied element-by-element with the underlying adjacency matrix Ak. This step generates the sample specific and dynamically tuned adjacency matrix A_cog,k_ simplified and shaped as (V, V). This dynamically generated A_cog,k_ is then used directly in the spatial graph convolution (GCN) module within the ST-GCN cell, which performs a graph convolution operation with the data input to the GCN module, followed by a temporal convolutional network module, and the result is summed up with the residual features, and finally passes through an activation function that constitutes the complete output of an ST-GCN cell. After the features have been extracted and abstracted layer by layer by such multiple dynamically tuned ST-GCN cells, the node dimensional information is aggregated by Global Average Pooling and finally fed into the Fully Connected Layer and Softmax function for action classification.

In order to verify the validity of the improvements of the CoG-STGCN model with respect to the baseline approach, we will detail the experimental setup, the dataset used, and the specific evaluation results in the next section.

## 4. Experiments and Result Analysis

### 4.1. Data Collection

Given the scarcity of public datasets for worker behavior on construction sites, we constructed a custom dataset focused on high-risk activities performed near edges to validate our proposed model. Drawing from the literature review [26,27,28,29] and field observations, we categorized high-frequency unsafe behaviors into three main types: movements with significant center of gravity (CoG) displacement, unstable postures, and distracted attention. Consequently, our dataset comprises six unsafe actions: running, jumping, quick squat, getting up quickly, squatting (long-time), and phone-walking. For contrast, two common safe behaviors, standing and walking, were also included. A detailed description of each action is provided in Table 4.

**Figure 8:**
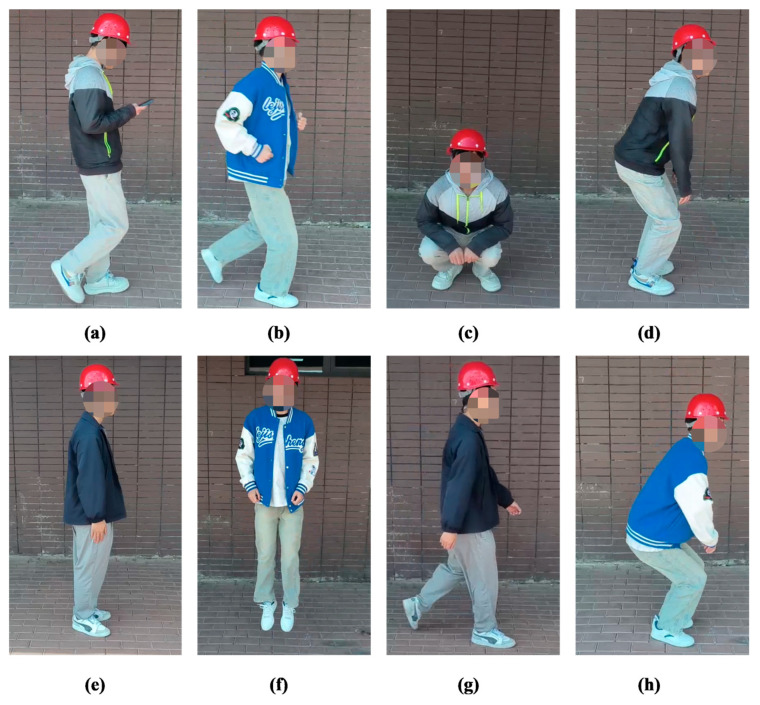
Illustrative examples of action samples. (**a**) phone-walking; (**b**) running; (**c**) squatting; (**d**) Getting up Quickly; (**e**) standing; (**f**) jumping; (**g**) walking; (**h**) quick squat.

To create the dataset, we recruited 10 volunteers, each performing 6 repetitions of the 8 specified actions. The actions were recorded using a standard mobile phone camera (1080 × 1920 resolution, 30 fps, OnePlus Ace 2, OnePlus, Shenzhen, China) and subsequently segmented into individual clips. This process yielded a total of 480 raw video clips, with an average duration of 3.42 s per clip. Care was taken to ensure that the subject’s keypoints remained unobstructed throughout each video. Illustrative examples of the collected action samples are shown in Figure 8. The clips for each action class were organized into separate folders to facilitate balanced data splitting.

To ensure a robust evaluation of our model’s generalization capability, we employed two distinct experimental protocols:

1. Fixed Train-Validation-Test Split: This protocol was used for initial model development and hyperparameter tuning. The 480 video clips were first partitioned into a training set (384 clips, 80%), a validation set (48 clips, 10%), and a test set (48 clips, 10%). Subsequently, data augmentation techniques, including horizontal flipping and random scaling, were applied exclusively to the training set, expanding it from 384 to 1152 samples. This strategy provides a consistent and static test bed for comparing model performance during the development phase.
2. Five-Fold Cross-Validation: To obtain a more reliable and generalized assessment of the final model’s performance, we employed a five-fold cross-validation scheme. The entire dataset of 480 clips was randomly partitioned into five equal, non-overlapping folds. In each of the five iterations, four folds (384 clips) were used for training, while the remaining fold (96 clips) served as the validation set. Similar to the fixed split, the training data in each iteration were augmented to 1152 samples. The final performance metrics were then averaged across all five folds. This approach minimizes the potential bias from a single, arbitrary data split.

For both protocols, the YOLOv8-Pose model was utilized to extract 2D skeleton sequences from the video frames. After processing, the skeleton data were saved as NumPy array files, and the corresponding labels were stored in pickle files, creating the required input format for the CoG-STGCN and ST-GCN models.

### 4.2. Validation of CoG Calculation Effectiveness

A core component of the proposed CoG-STGCN model is the computation of the body’s center of gravity from 2D keypoints. Therefore, validating the effectiveness of our weighted-average computation method is crucial. Considering that it is difficult to directly quantify the “real” center of gravity, this paper chooses to indirectly verify the validity of the method from the following three aspects, taking into account the feasibility and readability:

1. Comparison of CoG position with biomechanical reference points in static standardized posture

**Figure 9:**
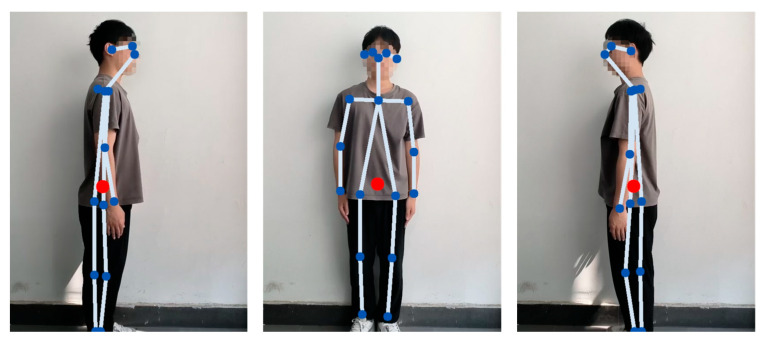
Example of calculated center of gravity position in standard standing position. The blue dots represent the detected skeleton keypoints, while the red dot indicates the computed center of gravity (CoG).

To initially assess the reasonableness of our weighted average CoG calculation method, we first examined the location of the center of gravity calculated in a standard static upright posture. According to widely accepted human biomechanical studies, the whole-body center of gravity of an adult in natural standing is located roughly within the pelvic region, approximately anterior to the second sacral vertebrae (S2). As shown in Figure 9, the location of the center of gravity computed by our weighted-average method is illustrated in a standard standing posture (red dots). As can be visually observed from the figure, the calculated center of gravity point indeed falls near the lower part of the torso of the skeleton, near the center region of the line connecting the two hip joints. Although the calculation based on 2D skeleton points and average segmental weights could not be precisely localized to the S2 vertebrae, its projected position in the image is consistent and as expected with the approximate region of the center of gravity described in the biomechanical literature. This provides a preliminary rationalization to support our CoG calculation methodology.

2. Stability observation of CoG trajectories in dynamic processes

**Figure 10:**
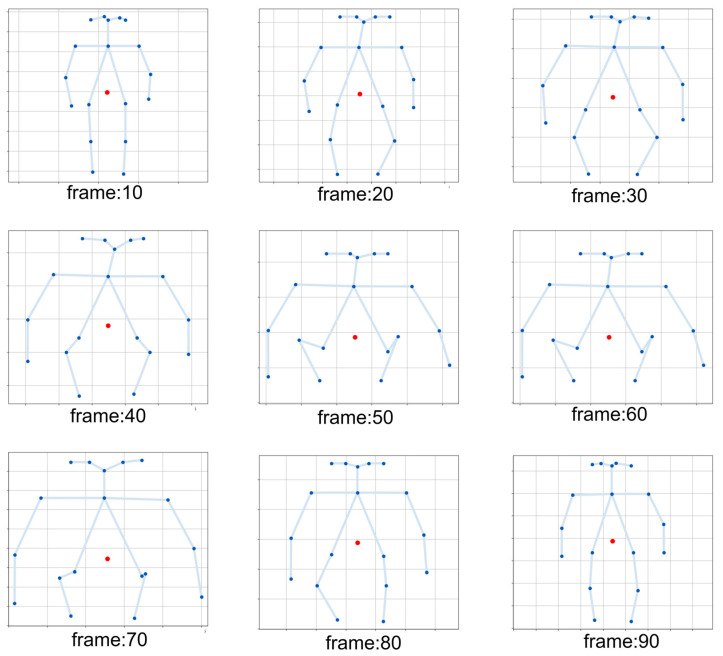
Schematic diagram of the change in the center of gravity position in different key frames during the rapid squat-to-stand process.

We validate the reasonableness and dynamic stability of the CoG computation strategy through a skeleton data visualization model that includes the center of gravity computation. We visualize a process of fast squatting to standing up to observe the dynamic trajectory of CoG, as shown in Figure 10. Frames 10 to 50 show the process of fast squatting, and it can be seen that the CoG point (the red origin) moves smoothly down along a near-vertical path, which is always kept in the double-legged position. The 60 to 90 frames show the process of squatting to standing, and the CoG point moves smoothly upward along a similar path until it returns to the initial standing height. Throughout the dynamics, the trajectory of the CoG is continuous and consistent with the biomechanical expectations of the maneuver, with no unreasonably violent oscillations. The reasonableness of this dynamic trajectory further strengthens our confidence in the validity of the adopted CoG calculation method.

3. Positive Impact on Downstream Behavior Recognition Task Performance

In addition to the above observations at the static and dynamic levels, the ultimate effectiveness of the center of gravity computation and its dynamic graph mechanism will be demonstrated by its practical contribution to the performance of downstream behavior recognition tasks. We expect that by introducing this biomechanically based center of gravity sensing capability, the CoG-STGCN model should be able to more accurately capture balance- and stability-related motion features compared to the original ST-GCN model with a static graph structure, and thus exhibit superior performance in recognizing specific risky behaviors. A detailed performance comparison and in-depth analysis of CoG-STGCN versus ST-GCN on our constructed proximity hazardous behavior dataset will be fully elaborated in Section 4.4.

### 4.3. Experimental Setup and Model Training

Validating the performance of the proposed model, this paper presents detailed training and testing validation of the CoG-STGCN model. The experiments are performed on a GPU equipped with NVIDIA GeForce RTX 4060 Ti, 16 GB video memory (NVIDIA Corporation, Santa Clara, CA, USA). The platform is powered by Ubuntu 20.04 LTS with a kernel version 5.4.0-155-generic operating system, and is configured with an Intel(R) Xeon(R) CPU E5-2683 v4 processor (Intel Corporation, Santa Clara, CA, USA) and 20 GB of system RAM. The development, training, and evaluation of the models were carried out in the Python 3.8.10 environment using the PyTorch 1.12.0 + CUDA 11.3 deep learning framework, which provides stable and efficient computational support for the whole experimental process.

For the selection of training parameters, we choose the stochastic gradient descent (SGD) optimizer to optimize the parameters of the proposed CoG-STGCN model. The initial learning rate (base_lr) is set to 0.01, and a learning rate decay strategy is configured, i.e., the learning rate is adjusted at the 60th and 100th cycles (epochs) of the training process. The weight_decay coefficient was set to 0.0005, and the batch_size was 16. The model was trained for a total of 120 epochs.

### 4.4. Experimental Results and Analysis

In order to verify the effectiveness of our proposed improved model, we trained the CoG-STGCN model obtained after the improvement with the baseline ST-GCN model under the same experimental platform and conditions, and tested its performance with the test set. Five main evaluation metrics are chosen for the model: the precision rate measures the proportion of samples predicted by the model to be positively classified that are actually also positively classified; the recall rate measures the proportion of all samples that are actually positively classified that are successfully predicted by the model to be positively classified; F1 value, as the reconciled average of precision rate and recall rate, combines these two metrics and is commonly used to evaluate the model’s overall performance on unbalanced datasets; Top-1 value, as the reconciliation mean of precision rate and recall rate, is commonly used to evaluate the model’s performance on unbalanced datasets and the overall performance of the model on an unbalanced dataset; Top-1 accuracy measures the proportion of samples for which the action category with the highest probability of prediction by the model agrees with the true action label. The formulas for precision, recall, F1-score and Top-1 accuracy are shown in (14)–(17):

(14) $$\mathrm{Precision}\;=\frac{TP}{TP+FP}$$

(15) $$\mathrm{Recall}=\frac{TP}{TP+FN}$$

(16) $$F1=\frac{2\times Precision\times Recall}{Precision+Recall}\times 100\%$$

(17) $$\mathrm{Top}-1\;\mathrm{Accuracy}=\frac{TP+TN}{TP+TN+FP+FN}$$

**Figure 11:**
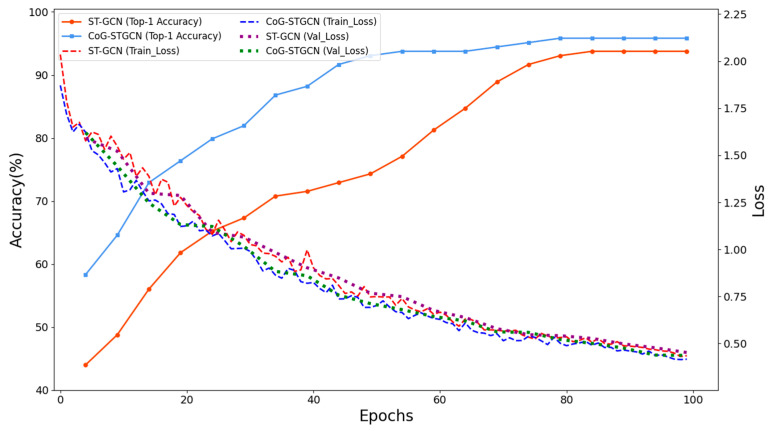
Top-1 accuracy and loss curves of ST-GCN and CoG-STGCN on the training and validation sets.

In this paper, the ST-GCN model and the improved CoG-STGCN model are used to train on the self-constructed behavioral dataset, and Figure 11 shows the accuracy curves of ST-GCN and CoG-STGCN on the validation set and the loss curves on the training set, with the accuracy and validation loss values being evaluated and recorded every five rounds (epochs), and the training loss values being recorded every round. Experimentally, the accuracy of the ST-GCN model (orange curve) shows rapid growth in the early training period (0–40 epochs), and then slows down and stabilizes at around 93% after about 80 epochs; in contrast, our improved CoG-STGCN generally outperforms the ST-GCN in terms of accuracy throughout the entire training cycle; specifically, the accuracy of CoG-STGCN is about 35% at the initial stage, the accuracy of CoG-STGCN is about 30% at the initial stage, and the accuracy of CoG-STGCN is about 30% at the initial stage. Specifically, the accuracy of CoG-STGCN rapidly climbs to over 90% in the first 35 epochs. In the 35–55 epoch phase, the accuracy rate enters a relatively flat growth plateau, which may be attributed to the fact that the model needs to additionally learn how to efficiently extract the four defined CoG features from the skeleton sequences while learning the basic spatio-temporal features, and the accuracy rate basically stabilizes around 95% after 70 epochs, indicating that the model has reached convergence.

Following the training phase, we evaluated the model performance on the test set. The results confirmed the superiority of our proposed model, which achieved an overall accuracy of 95.83%, surpassing the 93.75% accuracy of the original ST-GCN. To understand the contribution of each CoG-based feature, we conducted the ablation study shown in Table 5, revealing that all four features are beneficial since removing any single component decreases performance. The velocity-based features (f2 and f4) prove most critical, with the removal of global velocity (f2) causing the largest accuracy drop to 93.96%, indicating that dynamic motion intensity is the key discriminator our model learns. While spatial distance features (f1 and f3) also contribute, these results confirm that our method’s strength lies in leveraging dynamic cues from the CoG.

**Figure 12:**
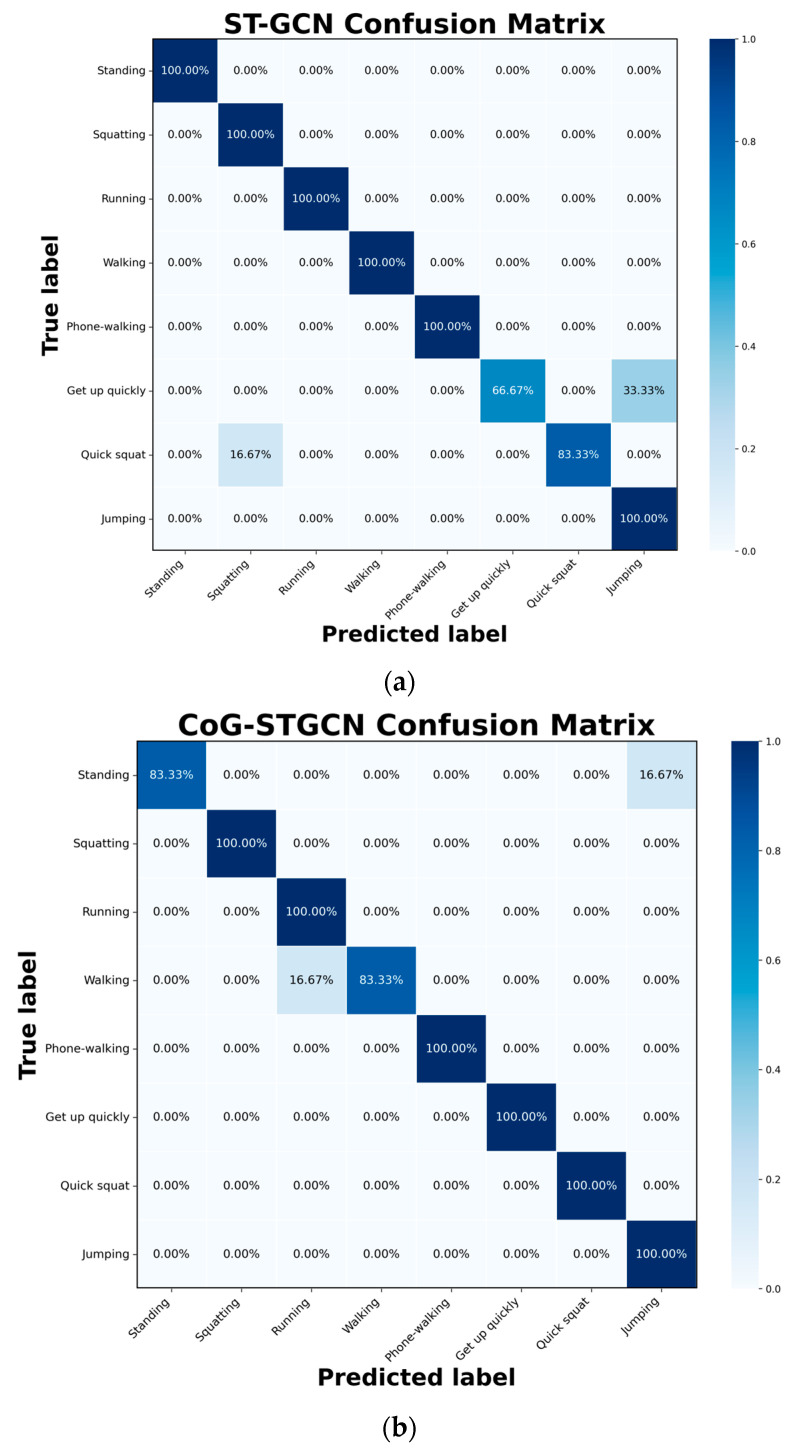
Mixing matrix of (**a**) ST-GCN and (**b**) CoG-STGCN on the test set.

An in-depth analysis of the classification performance for each action category was conducted, and confusion matrices were plotted to visually demonstrate the improvements over the baseline for both models, as shown in Figure 12. A detailed analysis of the confusion matrices reveals the specific advantages of our CoG-aware mechanism. For actions characterized by rapid and significant changes in the center of gravity, our CoG-STGCN model shows marked improvement. Specifically, for the ‘quick squat’ and ‘getting up quickly’ categories, CoG-STGCN achieves near-perfect recognition, whereas the baseline ST-GCN exhibits considerable confusion, misclassifying ‘getting up quickly’ as ‘jumping’ 33.33% of the time. This highlights that our CoG-based graph adjustment mechanism effectively captures the critical dynamic signatures of these high-risk movements, validating its design significance. However, the analysis also highlights certain limitations. For static actions like ‘standing’, the CoG features exhibit minimal variation, making it challenging for the MLP to learn effective adjustments. This leads to some confusion with ‘jumping’ in the CoG-STGCN model. Similarly, for periodic movements like ‘walking’, the subtle dynamic differences between it and ‘running’ are not fully captured by the current CoG features, resulting in some misclassification. This indicates that while our method excels in scenarios where CoG dynamics are a key discriminant, its universality is constrained by the expressive power of the selected features. Therefore, our model is particularly well-suited for applications focused on identifying movements with pronounced instability.

To further validate the robustness of the performance of the proposed CoG-STGCN model and reduce the chance of a single data division, we implemented a five-fold cross-validation of the CoG-STGCN and baseline ST-GCN models. Specifically, we randomly disrupted the overall dataset and divided it equally into five disjoint subsets. In each fold, one subset was selected as the validation set in turn, and the remaining four subsets were merged and processed with data enhancement to match the size of the training set in the previous experiments as the training set. Finally, we calculate and compare the mean and standard deviation of the key evaluation metrics of the two models in the five-fold cross-validation, and the results are shown in Table 6. In terms of the average accuracy, CoG-STGCN reaches 94.17%, which is about 1.26 percentage points higher than that of ST-GCN at 92.91%. Similarly, CoG-STGCN (94.27%) outperforms ST-GCN (93.03%) in terms of macro-averaged F1 scores for evaluating comprehensive multi-class categorization performance. Notably, the low standard deviation for both models indicates that their performance is not overly sensitive to the specific data partition, demonstrating good stability. Crucially, CoG-STGCN maintains this stability while achieving a higher level of average performance. Together, these results suggest that the introduction of the center-of-gravity-aware dynamic graph mechanism not only improves the average recognition ability of the model, but that this improvement is robust and credible.

To further evaluate the generalization ability of the CoG-STGCN model, we chose to test our model on the publicly available dataset Kinetics 400 dataset [30], which is a large-scale, high-quality human behavior recognition dataset published by DeepMind, containing approximately 300,000 video clips covering 400 different human action categories. We use the Kinetics-400 skeleton data available in the community, which was extracted from the original videos using OpenPose. The skeleton data contain 18 human body joints with node definitions consistent with our model format. Table 7 demonstrates the comparison of our model with other models. Our model achieves 33.1% in Top-1 accuracy, which is a 2.4% improvement compared to the 30.7% of ST-GCN, and this performance improvement is achieved while the number of parameters and computational effort of the model remain basically unchanged, as our model does not make any modifications to the convolutional network; compared to the MS-G3D model, our model has approximately 49.1% of its number of parameters and 69.2% of its computational effort. It can be seen that our improvement improves the performance of the benchmark model while maintaining the lightness of the model better.

### 4.5. Application Demonstration and Discussion

**Figure 13:**
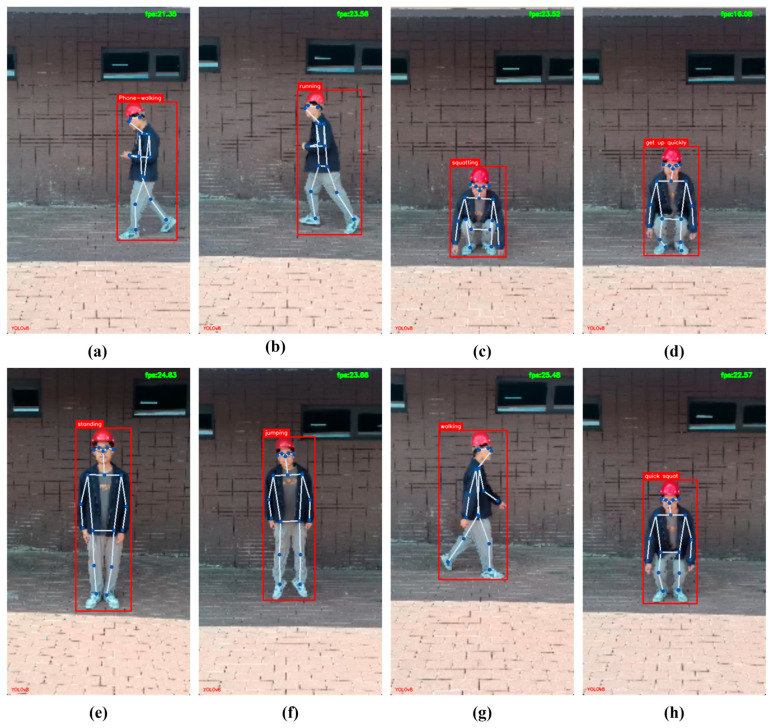
Visualization examples of recognition results. (**a**) Phone-walking; (**b**) running; (**c**) squatting; (**d**) getting up quickly; (**e**) standing; (**f**) jumping; (**g**) walking; (**h**) quick squat. Please note that the apparent low resolution of the visualized outputs is a result of the real-time processing pipeline, which prioritizes computational efficiency and speed over graphical quality.

In this study, a simple real-time behavior recognition system is designed, which is able to acquire real-time video streaming data from an RTSP protocol camera, extract the skeleton data sequence from continuous video frames via YOLOv8-POSE, and transmit the skeleton sequence to CoG-STGCN for behavior recognition and display the behavioral categories on top of the detection frames, and Figure 13 illustrates the final visualized output of the system, which contains the original video with the skeleton and detection frame superimposed. This system not only validates the feasibility of our proposed complete technological process from video input to behavioral categorization, but also provides an initial prototype for subsequent deployment and testing in real building environments.

To address the practical challenge of imperfect data, our CoG-STGCN model incorporates a robustness mechanism for handling partial occlusions or noisy detections. During inference, the system evaluates the confidence scores of keypoints required for CoG calculation. If an insufficient number of keypoints meet a predefined confidence threshold (e.g., 0.3), indicating unreliable input, the CoG-aware dynamic module is temporarily bypassed. In these instances, the model defaults to using only its base, fixed adjacency matrix, effectively reverting to the behavior of the original ST-GCN. The standard ST-GCN architecture implicitly handles missing joints by treating them as zero-value inputs and leveraging graph convolutions to aggregate features from visible neighboring joints. This trained ability to extract robust patterns from partial data ensures our system does not fail. By adopting this fallback strategy, our model maintains a baseline performance and enhances its overall robustness for real-world deployment where occlusions are common.

Furthermore, to address the practical concern of computational efficiency for potential real-world deployment, we conducted a preliminary performance analysis of our end-to-end system. Our benchmark tests on the experimental platform (NVIDIA GeForce RTX 4060 Ti) revealed that the proposed CoG-aware module introduces a minimal inference overhead to the overall pipeline. Specifically, a baseline system using YOLOv8s-Pose and the standard ST-GCN achieved an average processing speed of approximately 23.5 FPS. Our complete pipeline incorporating the CoG-STGCN model was found to run at approximately 23.1 FPS. This indicates that the added latency for CoG computation and dynamic graph modulation is only around 0.7 ms per inference cycle. While a comprehensive evaluation on actual edge devices is a crucial direction for future work, this result confirms that our method improves recognition accuracy at a negligible computational cost—a throughput reduction of less than 2%—thereby demonstrating its strong potential for real-time applications.

## 5. Conclusions

In this study, we addressed the critical challenge of identifying fall-risk behaviors on construction sites by proposing a novel center-of-gravity-aware spatio-temporal graph convolutional network, termed CoG-STGCN. Our core contribution is the explicit integration of center of gravity (CoG) dynamics—a key physical prior for human stability—into the construction of the graph, enabling the model to dynamically adapt to stability changes in real time. Experimental results on our self-constructed dataset of hazardous edge-related behaviors demonstrate the superiority of our approach. The CoG-STGCN achieved a Top-1 accuracy of 95.83% on the test set, outperforming the baseline ST-GCN (93.75%). Furthermore, it yielded a robust average accuracy of 94.17% in a five-fold cross-validation, a significant improvement over the baseline’s 92.91%. Notably, the performance gains were most pronounced in recognizing actions characterized by rapid CoG shifts, such as “quick squat” and “getting up quickly”.

These findings lead to a key insight: explicitly modeling the physical principles of human balance, rather than solely relying on learned feature-level correlations, is a more effective strategy for recognizing instability and potential fall risks. The success of CoG-STGCN validates that a physically informed model is more sensitive and interpretable, providing a new paradigm for developing more intelligent and proactive safety monitoring systems in the construction industry. By combining this approach with an efficient front-end like YOLOv8-POSE, our work presents a lightweight yet powerful scheme suitable for practical deployment.

Despite the promising results, this study has several limitations that open avenues for future research. A primary limitation is the scale and diversity of our self-constructed dataset. While sufficient to validate the core concept of CoG-STGCN, its generalization capability to complex, real-world construction environments with varied participants and conditions remains to be further verified. Consequently, the model’s robustness against severe occlusions and noisy detections, which are prevalent on actual sites, was not exhaustively evaluated. Future work will therefore prioritize the expansion and diversification of the dataset by recruiting more participants, collecting data from live construction sites to include naturalistic occlusions and noise, and employing advanced data augmentation techniques. Additionally, we plan to further enhance the model by exploring the fusion of multimodal information (e.g., 3D pose and environmental data) and validating its performance on edge devices.

## Figures and Tables

**Table 1:** Performance comparison with human pose estimation models on the COCO val2017 dataset.

Model	Input Size (Pixels)	AP (%)	AP@50 (%)	Params (M)	FLOPs (G)
Bottom-Up					
OpenPose (VGG19) [9]	-	61.8	84.9	49.5	221.4
HigherHRNet (HRNet-W32) [10]	512 × 512	67.1	86.2	28.6	47.9
Top-Down					
AlphaPose (ResNet-50) [15]	320 × 256	73.3	89.2	28.1	26.7
Mask R-CNN (ResNet-50-FPN) [17]	-	67.0	87.3	42.3	260
One-stage					
YOLOv8s-pose	640 × 640	59.2	85.8	11.6	30.2

**Table 2:** Global center of gravity node selection and weight assignment.

Body Segment	Keypoint Index	Weight
Head	0	0.08
Trunk	1, 11, 8	0.44
Left Arm	5	0.07
Right Arm	2	0.07
Left Leg	11	0.17
Right Leg	8	0.17

**Table 3:** Core area delineation and node selection.

ID	Core Region	Keypoint Index
*l*_1_	Head	0, 14, 15, 16, 17
*l*_2_	Trunk	1, 8, 11
*l*_3_	Left Arm	5, 6, 7
*l*_4_	Right Arm	2, 3, 4
*l*_5_	Left Leg	11, 12, 13
*l*_6_	Right Leg	8, 9, 10

**Table 4:** Descriptions of the eight edge-related action categories selected in the experiment.

Category	Action	Description
Significant CoG Displacement	Running	Large inertia, difficult to stop or turn quickly.
	Jumping	High impact upon landing, poor stability.
	Quick Squat	Rapid CoG drop, prone to dizziness or instability.
	Getting up Quickly	Rapid CoG rise, prone to dizziness or instability.
Unstable Posture	Squatting	Legs prone to fatigue, reduced coordination when standing up.
Distracted Attention	Phone-walking	Complete disregard for frontal and lateral road conditions.
Safe Behaviors	Standing	CoG stable, strong stability.
	Walking	Small CoG displacement, wide field of view.

**Table 5:** Ablation study results on CoG-STGCN components.

Methods	Accuracy (Top-1)	Comparison with Baseline
ST-GCN(Baseline)	93.75%	-
CoG-STGCN (f1 + f2 + f3 + f4)	95.83%	+2.08%
w/o Dist_GlobalCoG (f1)	95.21%	+1.46%
w/o Vel_GlobalCoG (f2)	93.96%	+0.21%
w/o Dist_CoreCoG (f3)	94.79%	+1.04%
w/o Vel_CoreCoG (f4)	94.17%	+0.42%

**Table 6:** Performance comparison of ST-GCN and CoG-STGCN using five-fold cross-validation on the self-collected dataset.

Model	Metric	Cross Validation					Average ± Std Dev
		1	2	3	4	5	
ST-GCN	Accuracy	0.9270	0.9479	0.9062	0.9375	0.9270	0.9291 ± 0.0146
	Macro-F1	0.9283	0.9491	0.9075	0.9384	0.9281	0.9303 ± 0.0146
CoG-STGCN	Accuracy	0.9375	0.9583	0.9167	0.9583	0.9375	0.9417 ± 0.0169
	Macro-F1	0.9387	0.9594	0.9177	0.9592	0.9384	0.9427 ± 0.0169

**Table 7:** Performance and efficiency comparison of different action recognition models on the Kinetics-400 dataset.

Models	Accuracy (Top-1)	Accuracy (Top-5)	Params (M)	FLOPs (G)
ST-GCN [5]	30.7%	52.8%	3.10	16.30
2s-AGCN [21]	36.1%	58.7%	6.94	37.30
Deep LSTM [5]	16.4%	35.3%	-	-
MS-G3D [22]	38.0%	60.9%	6.44	24.50
CTR-GCN [23]	-	-	5.84	7.88
CoG-STGCN (ours)	33.1%	55.7%	3.16	16.95

## Data Availability

The raw data supporting the conclusions of this article will be made available by the authors on request.
